# Leveraging Fst and Genetic Distance to Optimize Reference Sets for Enhanced Cross-Population Genomic Prediction

**DOI:** 10.3390/ani16030359

**Published:** 2026-01-23

**Authors:** Le Zhou, Lin Zhu, Fengying Ma, Mingjuan Gu, Risu Na, Wenguang Zhang

**Affiliations:** 1 College of Animal Science, Inner Mongolia Agricultural University, Hohhot 010010, China; zxcvbnm8880314@163.com (L.Z.); zhulinynacxhs@163.com (L.Z.); fengyingma1997@163.com (F.M.); gmj0119@yeah.net (M.G.); narsanjargal@imau.edu.cn (R.N.); 2 Inner Mongolia Engineering Research Center of Genomic Big Data for Agriculture, Hohhot 010010, China; 3 College of Life Science, Inner Mongolia Agricultural University, Hohhot 010010, China

**Keywords:** genomic selection, cross-population prediction, genetic distance, fixation index (Fst), admixture proportion

## Abstract

Genomic selection often faces challenges of insufficient prediction accuracy in cross-population applications, primarily due to differences in linkage disequilibrium patterns between populations. This study proposes an Fst-based strategy to enhance prediction performance by constructing a cross-population reference set with high genetic similarity to the target population (PopA). By integrating Fst-mediated SNP screening and Euclidean genetic distance analysis, the top 10%, 15% and 20% of individuals genetically most similar to PopA were screened from PopB and PopC, respectively, leading to the generation of six reference sets characterized by different mixing proportions. The results demonstrate that incorporating the top 10–20% of the most similar individuals significantly improves the accuracy and robustness of genomic estimated breeding value predictions. Among the methods evaluated, ssGBLUP and wGBLUP performed best, with prediction accuracy increasing as the mixing proportion rose up to 20%. This approach effectively mitigates structural bias caused by inter-population genetic differences and significantly enhances prediction efficiency. The multi-level mixing experiment not only validates the practical value of Fst and Euclidean distance but also provides theoretical support and a feasible solution for the efficient integration of cross-population germplasm resources.

## Simple Summary

Genomic prediction across populations often suffers from low accuracy due to genetic differences. This study introduces an Fst-based approach to select individuals from other populations that are genetically similar to the target population. By including the top 10–20% most similar individuals, prediction accuracy and robustness were significantly improved, with methods like ssGBLUP performing best. This strategy helps reduce bias and enhances breeding efficiency across diverse populations.

## 1. Introduction

In the modern breeding field, accurate genetic evaluation serves as the fundamental cornerstone for achieving efficient genetic improvement and cultivating superior breeds. Through genetic evaluation, breeders can gain in-depth insights into the genetic merit of individuals, thereby enabling targeted selection of breeding stock with excellent genetic characteristics for reproduction—this targeted approach significantly enhances breeding efficiency and ensures the quality of improved varieties, directly addressing the core demand for high-performance breeds in practical production. Traditional genetic evaluation has long been confined within single breeds; however, with the global development of animal husbandry and agriculture, the genetic resources of individual breeds have become increasingly insufficient to meet the growing demands for improved productivity, adaptability, and quality [1,2,3]. As an innovative and practical strategy, cross-breed genetic evaluation breaks through the limitations of single-breed evaluation by integrating genetic information across multiple breeds, which not only facilitates the full excavation and utilization of abundant genetic diversity but also provides a feasible path for overcoming the bottleneck of limited genetic resources in traditional breeding. Its core importance lies in bridging the genetic gap between different breeds, while its practical applicability is reflected in its ability to directly support the development of high-performance breeds that meet diverse production needs, thus laying a solid foundation for the sustainable development of the breeding industry and highlighting the necessity and value of the present study.

As a key technology for integrating genetic resources of different breeds and accelerating breeding progress, cross-breed genetic evaluation has attracted numerous researchers to conduct extensive studies and propose various methods, which mainly fall into two mainstream technical pathways: one is the traditional genetic evaluation method based on pedigree information [4,5,6,7]; the other is the genomic selection method utilizing molecular markers [8,9,10]. Although these methods have improved the feasibility of cross-breed genetic evaluation to a certain extent, they still have significant limitations that severely restrict their application effects in practical breeding, highlighting the urgency of further optimizing technical solutions and the core value of this study. Specifically, a study by Clasen et al. [11] clearly pointed out that when individuals from each cattle herd are included in the reference population, the accuracy of genomic estimated breeding values (GEBV) mainly depends on intra-population linkage information; even if different breeds are merged to construct a reference population, the improvement in GEBV accuracy is still relatively limited—this means that the single-breed evaluation model is highly dependent on intra-population genetic information, and for breeds or populations not included in the reference population, the prediction accuracy will decrease significantly, making it difficult to meet the practical needs of cross-breed resource integration. On the other hand, traditional pedigree methods are limited by the accuracy and completeness of records, and perform poorly in evaluating genetic relationships between distantly related breeds: pedigree errors or missing data will directly affect the accurate inference of genetic background, while distantly related breeds themselves have significant genetic differences and low levels of linkage disequilibrium, further leading to the difficulty of traditional methods in reliably assessing their genetic associations [8,12]. Even though genomic selection methods have introduced genome-wide information, they still face major challenges in coping with complex genetic backgrounds and structural differences between breeds.

As a pivotal core technology in modern breeding practices, the prediction precision of GS is largely contingent on the genetic relatedness between the reference population and the target population [13]. In recent years, to improve cross-population prediction performance, many studies have attempted to use genetic correlations between populations to optimize the construction of reference populations. For example, Yin et al. [14] systematically evaluated the performance of various statistical models in multi-breed genomic prediction by simulating populations of different sizes. The study found that integrating data from multiple populations can significantly improve prediction accuracy, especially when heritability is high or there is a genetic relationship between populations. Chang et al. [15] adopted Fst-driven SNP prioritization and weighting approaches to build a functionally weighted G matrix, substantially augmenting the genetic affinity between training and validation populations. In comparison with the traditional equal-weight model, this strategy boosted genomic prediction accuracy by more than 5%, providing a new avenue for the efficient exploitation of high-density marker datasets in livestock genomic selection. These studies show that reasonably using cross-population genetic associations can improve the generalization ability of genomic prediction to some extent. However, the above methods still face important challenges. On the one hand, directly combining samples between populations with significant genetic background differences can easily introduce noise and reduce model estimation efficiency [16,17]. On the other hand, existing methods mostly rely on overall genetic similarity and fail to fully explore local genomic differentiation information related to the target trait, resulting in insufficient prediction specificity for specific traits [18].

Against this backdrop, the fixation index (Fst) has been widely recognized as a robust and reliable indicator for quantifying the degree of genetic differentiation between populations. It can effectively reveal differences in allele frequencies, selection pressures, and adaptive traits across populations, thereby providing a precise and actionable tool for identifying genome-wide highly differentiated regions and screening individuals with close genetic similarity—an essential foundation for addressing the core challenge of cross-population genetic evaluation [19,20]. Therefore, this study proposes a cross-population genetic evaluation strategy based on Fst-stratified screening. The aim is to identify individuals with the highest similarity to the target population (PopA) in different genetic backgrounds by calculating Euclidean genetic distances based on highly differentiated SNPs between populations. Specifically, we first screen for highly differentiated loci between PopA and PopB, PopC through Fst analysis. Based on this, we calculate individual-level genetic distances and select the top 10%, 15%, and 20% of individuals from PopB and PopC that have the highest genetic similarity to PopA. Further, we merge these individuals with the entire PopA population and systematically introduce corresponding proportions of individuals from PopC to construct six cross-population reference sets with different mixing ratios. This design allows us to comprehensively evaluate: (1) the impact of genetic distance thresholds (i.e., similarity screening ratios) on the composition of the reference population; (2) the effect of mixing ratios on the prediction accuracy of GEBVs; (3) the potential of the optimal cross-population construction strategy in balancing genetic diversity and prediction accuracy.

## 2. Materials and Methods

### 2.1. Data Simulation

We employed QMSim Version 1.10 [21] to simulate livestock genomic datasets, with the objective of exploring how distinct linkage disequilibrium (LD) patterns influence the prediction accuracy of GEBVs in beef cattle populations. The simulation resulted in three beef cattle populations (PopA, PopB, and PopC) with distinct LD patterns. Each population underwent five replicate simulations to generate five sets of final breeding animals. In this simulation, only the genotypes and phenotypes of generations 9 and 10 were generated. Phenotypes followed a normal distribution with a mean of 0 and variance of 1, and the overall phenotypic mean was governed by one fixed effect.

(1) Population A (PopA) was modeled to represent a linkage disequilibrium (LD) pattern that starts from a stable state and continues to expand. The historical population (HP) was initially set at 200 individuals, with this size sustained for 1000 generations. Thereafter, the population was scaled up to 1000 individuals (100 males and 900 females) over the next 95 generations, thereby establishing LD state and reaching mutation-drift equilibrium. From the terminal generation of the HP, 250 males and 3000 females were randomly chosen to initiate population expansion, with random mating performed for 10 generations (one offspring per female per generation). Subsequently, 50 males and 2000 females were randomly selected from the expanded cohort and simulated over the latest 100 generations, maintaining the same offspring production rate (one per female per generation). Selection was based on phenotypic performance and breeding values (EBVs) estimated via Best Linear Unbiased Prediction (BLUP), with replacement rates of 60% for bulls and 30% for cows, and a phenotypic record missingness rate of 5%.
(2) Population B (PopB) was simulated to reflect LD pattern that initially contracts and then expands. The initial population comprised 500 individuals, which underwent a size reduction to 200 individuals over 1000 generations and was subsequently expanded to 1000 individuals (100 males and 900 females) during the next 95 generations, for the purpose of establishing the initial LD pattern and reaching mutation-drift equilibrium. From the terminal generation of the HP, 250 males and 2550 females were randomly chosen to initiate population expansion, with random mating performed for 10 generations (one offspring per female per generation). Subsequently, 55 males and 2100 females were randomly selected from the expanded cohort and simulated over the latest 100 generations, maintaining the same offspring production rate (one per female per generation). Selection was based on phenotypic performance and EBV estimated BLUP, with replacement rates of 50% for bulls and 30% for cows, and a phenotypic record missingness rate of 5%.
(3) Population C (PopC) was modeled to simulate LD pattern that first expands and then contracts. The initial population comprised 1000 individuals, which underwent a size reduction to 200 individuals over 1000 generations and was subsequently expanded to 1000 individuals (100 males and 100 females) during the next 95 generations, for the purpose of establishing the initial LD pattern and reaching mutation-drift equilibrium. From the terminal generation of the HP, 250 males and 3000 females were randomly chosen to initiate population expansion, with random mating performed for 10 generations (one offspring per female per generation). Subsequently, 50 males and 2200 females were randomly selected from the expanded cohort and simulated over the latest 100 generations, maintaining the same offspring production rate (one per female per generation). Selection was based on phenotypic performance and EBV estimated BLUP, with replacement rates of 50% for bulls and 20% for cows, and a phenotypic record missingness rate of 5%.

For the present simulation, QMSim was configured to set initial parameters of LD, mutation, and drift under conditions including equal sex ratio, non-overlapping generations, random mating, lack of selection pressure, and no migration events, which enabled the mimicry of the inherent LD structure and extent characteristic of beef cattle populations [22]. The animals genotyped include all individuals from the 95th to the 100th generation. Individuals from the 95th to the 98th generations were randomly selected to form the reference population. Due to variations in the number of animals in the 99–100th generation across breeds, the number of genotyped individuals varied slightly for each breed. Table 1 presents a comprehensive summary of the population structure and key parameters employed in the simulation.

#### 2.1.1. Genome

To simulate the 29 autosomal chromosome pairs of beef cattle, we adopted the ARS-UCD1.2 bovine genome assembly [23], which spans a total genetic length of 2715.85 centimorgans. This setup enhanced the realism of the simulation by incorporating the actual genomic distances between molecular markers and quantitative trait loci (QTL). We generated 50 k single-nucleotide polymorphism (SNP) markers in a uniformly random distribution; these markers represented diallelic segregating loci, with the count of SNPs per chromosome scaled to the chromosome’s physical size, and were assumed to exert no phenotypic effects (neutral markers). A total of 725 QTL were distributed across the genome. Recombination events were modeled per Morgan via sampling from a Poisson distribution (mean = 1), with crossovers randomly positioned along each chromosome. In the terminal generation of the historical population (HP), we assigned genetic effects to a production trait with a heritability of 0.42—consistent with the reported heritability of birth weight in beef cattle [24]. Within QMSim, the genetic effects of this trait were drawn from a gamma distribution (shape parameter = 0.4), such that the total genetic variation was fully explained by the combined QTL effects. Over the 1095 generations of the HP, both QTL and SNPs were assigned a mutation rate of 2.5 × 10^−5^. SNP mutations were configured as “recurrent,” meaning allele transitions occurred only between existing variants (no novel alleles were generated). All simulation parameters are detailed in Table 1.

#### 2.1.2. Genomic Evaluation of Breeding Value

In the present simulation, the target trait was assigned a heritability of 0.42, with its phenotypic variance standardized to 1.0. TBV of each beef cattle individual was determined by summing the additive genetic effects of all QTLs, as indicated below:

(1) $${TBV}_{k}=\sum_{j=1}^{QTL}\beta_{j}\cdot Q_{kj},$$

where β_j_ represents the additive impact of the j-th QTL, Q_kj_ denotes the number of copies of a specific QTL variant present in individual k, which can be 0, 1, or 2. Phenotypic expressions (yi) were generated by incorporating a residual term drawn from a normal distribution N (0, $\sigma_{r}^{2}$ ), where $\sigma_{r}^{2}$ signifies the residual variance.

EBV were computed for every individual in the current cohort (generations 9 to 10) by leveraging both phenotypic performance data and pedigree relationship information. The breeding values with the lowest prediction error variance were obtained through the best linear unbiased prediction (BLUP) method from a mixed linear model [25]. The numerator relationship matrix (A) is incorporated into the following mixed-model equations to derive the BLUP of random additive effects, including polygenes and QTLs:

(2) $$\left[Z^{\prime }Z+A^{-1}\frac{\sigma_{e}^{2}}{\sigma_{a}^{2}}\right]\left[\hat{a}\right]=\left[Z^{\prime }y\right],$$

where y denotes the phenotypic observation vector, Z represents the incidence matrix associating phenotypic records with random additive genetic effects (a), $\sigma_{e}^{2}\;$ indicates the residual variance, and $\sigma_{a}^{2}$ denotes the additive genetic variance. Solutions for the mixed-model equations were derived using the conjugate gradient algorithm.

### 2.2. Scenarios

In this study, a cross-population reference set was constructed through Fst-mediated SNP screening, Euclidean genetic distance calculation, and a stratified mixing strategy. The specific steps are as follows:

1. Screening of highly differentiated SNPs: First, pairwise fixation index (Fst) values between the target population (PopA) and candidate reference populations (PopB, PopC) were calculated. Using Fst > 0.1 as the threshold, highly differentiated SNPs between populations were screened to provide a core marker basis for subsequent genetic similarity assessment;
2. Calculation of Euclidean genetic distance: An individual genetic similarity matrix was constructed based on the genotypic data of the screened highly differentiated SNPs. The genetic distance between each individual in PopB/PopC and individuals in PopA was defined as the Euclidean distance between genotype vectors, quantifying the genetic similarity at the individual level across populations;
3. Screening of highly similar individuals: Individuals in PopB and PopC were ranked by Euclidean distance in ascending order. The top 10%, 15%, and 20% of individuals with the highest genetic similarity to PopA were selected from PopB and PopC, respectively, forming subsets of highly similar individuals with different proportions;
4. Construction of cross-population reference sets: A stratified mixing strategy was adopted. The subsets of highly similar individuals with different proportions screened from PopB and PopC were combined with the original reference individuals of PopA, respectively, to generate 6 cross-population reference sets specific combinations:
  (1) A + 10%B: Core population A supplemented with individuals showing the top 10% genetic distance from population B;
  (2) A + 15%B: Core population A supplemented with individuals showing the top 15% genetic distance from population B;
  (3) A + 20%B: Core population A supplemented with individuals showing the top 20% genetic distance from population B;
  (4) A + 10%C: Core population A supplemented with individuals showing the top 10% genetic distance from population C;
  (5) A + 15%C: Core population A supplemented with individuals showing the top 15% genetic distance from population C;
  (6) A + 20%C: Core population A supplemented with individuals showing the top 20% genetic distance from population C.

By systematically evaluating the genomic prediction performance under different combinations of genetic distance thresholds and mixing proportions, this study aims to reveal the optimal population structure and individual introduction strategy in cross-population genetic evaluation, thereby providing a theoretical basis and scheme reference for improving the accuracy of genomic estimated breeding value (GEBV) prediction.

### 2.3. Model and Analysis

The genomic prediction models employed in this study included Genomic Best Linear Unbiased Prediction (GBLUP), Single-Step Genomic Best Linear Unbiased Prediction (ssGBLUP), and weighted Genomic Best Linear Unbiased Prediction (wGBLUP), which were utilized for estimating Genomic Estimated Breeding Values (GEBV). The selection of these models was based on three core considerations. First, these three models are widely applied in cross-population genomic selection studies, which ensures the practical reference value of the research results. Second, they exhibit distinct statistical principles: GBLUP adopts equal weights for SNP effects, ssGBLUP can integrate both pedigree and genomic data, and wGBLUP is capable of weighting SNP effects. These differences allow for a systematic comparison of the adaptability of different model frameworks to the cross-population reference sets constructed in this study. Third, the models possess feasibility for breeding practice. In particular, ssGBLUP and wGBLUP have been widely used in commercial breeding scenarios due to their ability to handle incomplete genotypic data and improve the prediction accuracy of complex traits. By evaluating the prediction performance of the three models across different cross-population reference sets, the universality of the reference set construction strategy proposed in this study can be verified, thereby enhancing the robustness and practical guiding significance of the research results.

For the estimation of parameters in GBLUP using genomic data, a general linear mixed model is applied. This model was constructed using the HIBLUP_1.4.0 software package. While both GBLUP and PBLUP models incorporate identical fixed effects, GBLUP employs a genomic relationship matrix (GRM) derived from SNP markers to estimate GEBV. The formula for calculating GRM is provided below:

(3) $$G=\frac{MM^{\prime }}{\sum_{i=1}^{m}{2p}_{i}(1-p_{i})},$$

where M represents the matrix of individual gene counts (with homozygotes, heterozygotes, where the two types of homozygotes and the heterozygote are coded as 0, 1, and 2, respectively; m refers to the total number of SNP markers; and p_i_ stands for the allele frequency at the i-th SNP locus.

Given that the model was restricted to the inclusion of additive genetic effects only, these effects are accounted for as follows:

(4) $$Var\left(a\right)=G\sigma_{g}^{2},$$

The full linear mixed-model equation for GBLUP is presented as follows:

(5) y=Xb+Za+e,

(6) $$\left[\begin{array}{cc} X^{\prime }X & X^{\prime }Z\\ Z^{\prime }X & Z^{\prime }Z+\lambda G^{-1} \end{array}\right]\left[\begin{array}{c} \hat{b}\\ \hat{g} \end{array}\right]=\left[\begin{array}{c} X^{\prime }y\\ Z^{\prime }y \end{array}\right],$$

In these equations, y denotes the vector of phenotypic observations; X represents the design matrix relating fixed effects to each individual; b is the vector of fixed effects; Z denotes the design matrix assigning phenotypic records to corresponding genetic values; g is the vector of additive genetic effects for all individuals; G refers to the genomic relationship matrix; and e is the vector of residual error effects that follows a normal distribution, ~N(0, $G\sigma_{e}^{2}$ ), $\sigma_{e}^{2}$ is the residual variance and $\lambda =\sigma_{e}^{2}/\sigma_{g}^{2}$ .

For the ssGBLUP method, its statistical framework is analogous to that of traditional genetic assessment approaches. However, this method distinctively incorporates both non-genotyped and genotyped animals into a composite relationship matrix H. This matrix H integrates the A matrix, which represents the numerator relationship, with the G matrix, which denotes the genomic relationship. In this context, the inverse of A^−1^, is substituted with the inverse of H^−1^ as developed by Aguilar et al. [26], and its mathematical expression is outlined as follows:

(7) $$H^{-1}=A^{-1}+\left[\begin{array}{cc} 0 & 0\\ 0 & G^{-1}-A_{22}^{-1} \end{array}\right],$$

where $H^{-1}$ is the inverse of the matrix combining pedigree and genomic data, $G^{-1}$ is the inverse of the genomic relationship matrix, and $A_{22}^{-1}$ refers to the inverse of the numerator relationship matrix for genotyped animals. The G matrix was constructed using the formula outlined in Equation (3).

For wGBLUP, the basic model and inference process remain consistent with conventional approaches, with the primary distinction being the incorporation of a distinct SNP effect vector in the construction of the G matrix. Utilizing the SLEMM software (v0.90.1) [27], two strategies for refining genomic prediction through SNP weighting are implemented: (1) a method that accounts for the minor allele frequency (MAF) influence on SNP effect magnitudes; (2) an approach that leverages SNP effect estimates with weights W set to the identity matrix.

SLEMM accommodates the following linear mixed model:

(8) $$\begin{array}{c} y=X\beta +Z\alpha +e\\ \alpha \sim N(0,W\sigma_{\alpha }^{2}),\\ e\sim N(0,R\sigma_{e}^{2}) \end{array}$$

where y is the vector of quantitative trait phenotypes; β includes fixed effects (such as the mean); X is the design matrix for β; α is the vector of SNP effects with a diagonal covariance matrix $W\sigma_{\alpha }^{2}$ ; Z is the standardized genotype matrix; and e is the residual vector with a diagonal covariance matrix $R\sigma_{e}^{2}$ . The diagonal elements of W are weights, where each weight signifies the relative impact of the corresponding SNP on genetic variance, i.e., W_j_ indicates the SNP j’s contribution to genetic variance.

Given the tendency for nearby SNP loci to capture the effects of a quantitative trait locus (QTL) due to linkage disequilibrium (LD), and the similarity in the effects of adjacent SNPs in model fitting, this study employs the second SNP weighting scheme, defined as:

(9) $$W_{jj}=C\cdot \frac{1}{2S+1}\sum_{k-j-S}^{j+S}\hat{\alpha_{k}^{2}},$$

where C is a scaling constant that ensures the mean weight equals 1; S is the number of SNPs on either side of SNP j; and $\hat{\alpha_{k}}$ is the estimated effect of the k-th SNP from a pre-fit BLUP model with W as the identity matrix. The weight of each SNP is derived from a neighboring window containing 2S + 1 SNPs. SLEMM is implemented in two steps: first, fitting the model to training data with W as the identity matrix, then refitting the model using W computed via Equation (9).

### 2.4. Genomic Prediction Accuracy

In this research, the efficacy of the model was gauged through the metrics of predictive precision and fairness. Given that TBV were explicitly given in the simulation, predictive precision is quantified as the Pearson correlation between the genomically GEBV and TBV, as follows:

Accuracy = Corr (GEBV, TBV),(10)

where the correlation coefficient ranges from 0 to 1, reflecting the degree of linear association between GEBV and TBV.

Furthermore, the fairness of the predictions is evaluated by the regression coefficient of GEBV on TBV, determined by:

b = Cov(GEBV, TBV)/Var(GEBV),(11)

When b approximates 1 and the intercept is close to 0, the genomic predictions are deemed impartial.

### 2.5. Two-Way Analysis of Variance (2-Way ANOVA)

To statistically evaluate the differences in predictive accuracy across different populations and conditions, we conducted a two-way analysis of variance (2-way ANOVA) using Prism software (v8.0.2). This method allows us to examine the main effects of two categorical independent variables, as well as their potential interactive effects, on a continuous dependent variable—in our case, genomic prediction accuracy. The two-way ANOVA model included two factors: (1) the impact of different admixed populations (e.g., PopB and PopC) on genomic prediction accuracy; (2) the influence of condition factors (e.g., different mixing proportions, evaluation models) on genomic prediction accuracy. The two-way ANOVA provided estimates of the main effects for each factor, as well as the interaction effect between the two factors. The significance of these effects was determined by the calculated F-statistics and the corresponding *p*-values. A *p*-value less than 0.05 was considered to indicate a statistically significant difference.

## 3. Results

### 3.1. Population Genetic Characterization

**Figure 1:**
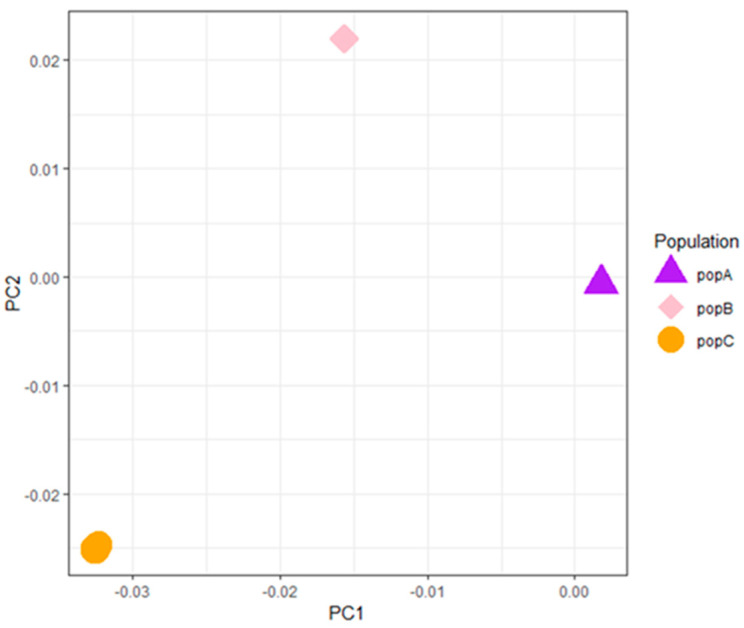
Principal Component Analysis (PCA) results for three different populations (PopA, PopB, and PopC). The x-axis represents Principal Component 1 (PC1), and the y-axis represents Principal Component 2 (PC2). Different colors and shapes of points represent different populations, where purple triangles represent PopA, pink diamonds represent PopB, and orange circles represent PopC.

To investigate population structure, we conducted a Principal Component Analysis (PCA). As shown in Figure 1, the three populations (PopA, PopB, and PopC) are distinctly separated on the first and second principal components (PC1 and PC2). The clear clustering of PopA, PopB, and PopC indicates significant genetic differentiation between them.

**Figure 2:**
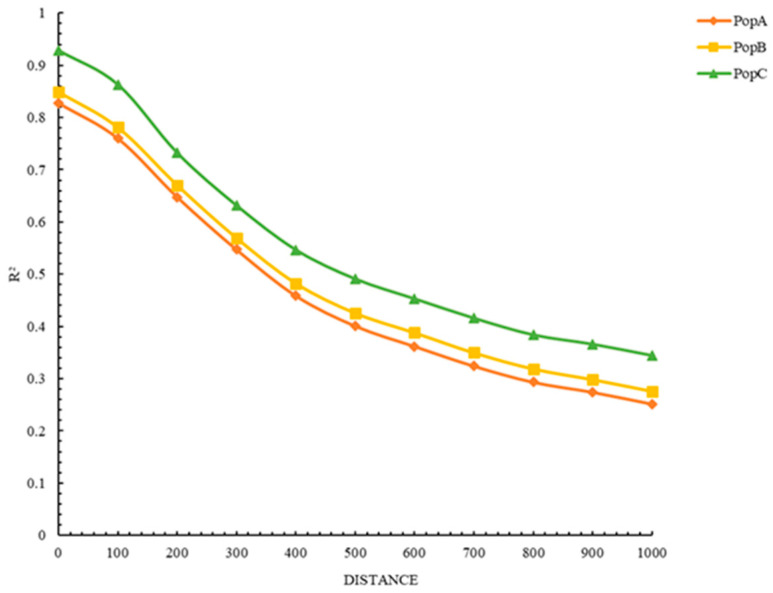
LD among markers in the 10th (most recent) generation of three distinct populations (PopA, PopB, and PopC). The x-axis denotes the inter-marker genomic distance range (units: kilobases, kb), and the y-axis represents the average LD quantified by the squared correlation coefficient (R^2^).

We carried out linkage disequilibrium (LD) decay analysis to assess the extent of LD changes with increasing physical distance. LD refers to the non-random association between the allele frequencies of two or more loci in the genome. As shown in Figure 2, the LD decay curves indicate that the LD values (measured as R^2^) in all three populations decline as the distance between genes increases. However, the rate of LD decay varies among populations, consistent with the LD decay trends reported in the literature. Specifically, PopC has the slowest LD decay rate, meaning that the non-random association between alleles at different loci can persist over longer physical distances in this population. In contrast, PopA has the fastest LD decay rate, indicating that genetic linkage between loci is more rapidly disrupted with increasing distance. For all three populations, the LD (R^2^) values gradually decrease as the physical distance between markers extends. This consistent downward trend across populations suggests that the strength of genetic linkage weakens as the physical distance between genomic loci increases.

**Figure 3:**
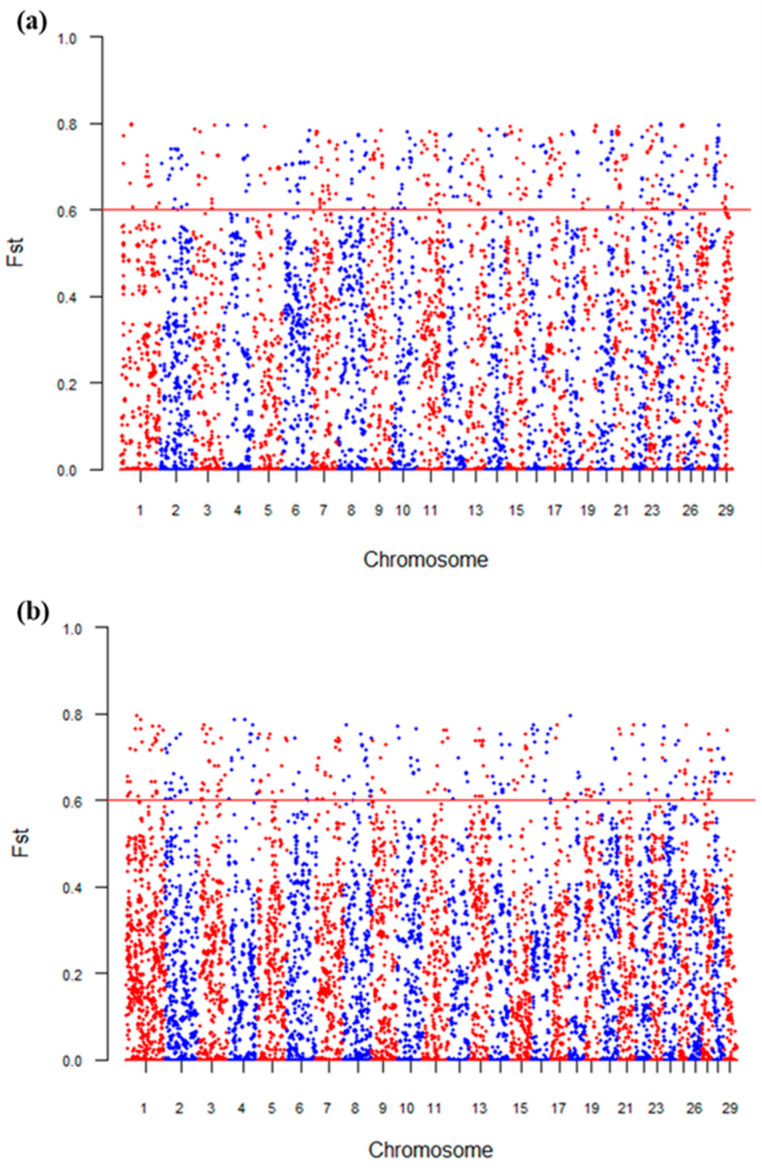
Manhattan plots of genetic differentiation between PopA and PopB/PopC based on Fst. (**a**) Manhattan plot of genetic differentiation between PopA and PopB; (**b**) Manhattan plot of genetic differentiation between PopA and PopC. The x-axis shows the chromosome number; the y-axis shows the Fst value of SNPs, with the red horizontal line indicating the threshold for strong differentiation (0.6).

We plotted a Manhattan plot of the fixation index (Fst) to identify genomic regions with high genetic differentiation. As shown in Figure 3, numerous single-nucleotide polymorphisms (SNPs) across different chromosomes exhibit varying Fst values. SNPs with Fst values exceeding the red horizontal threshold line indicate genomic regions with strong genetic differentiation, suggesting that these regions may be under selection or other evolutionary forces. Specifically, the Fst values between PopA and PopB are mostly concentrated between 0.1 and 0.3, with a few exceeding the threshold (Figure 3a). In contrast, the Fst values between PopA and PopC are generally higher, with a significant increase in the number of sites exceeding the threshold (Figure 3b). The overall pattern of high differentiation sites is characterized by “continuous clusters,” indicating more extensive and concentrated genetic differentiation compared to that between PopA and PopB.

### 3.2. Comparative Analysis of Prediction Accuracy of Single Population and Cross-Population Under Different Genetic Distance Levels Using Three Genomic Prediction Methods

**Figure 4:**
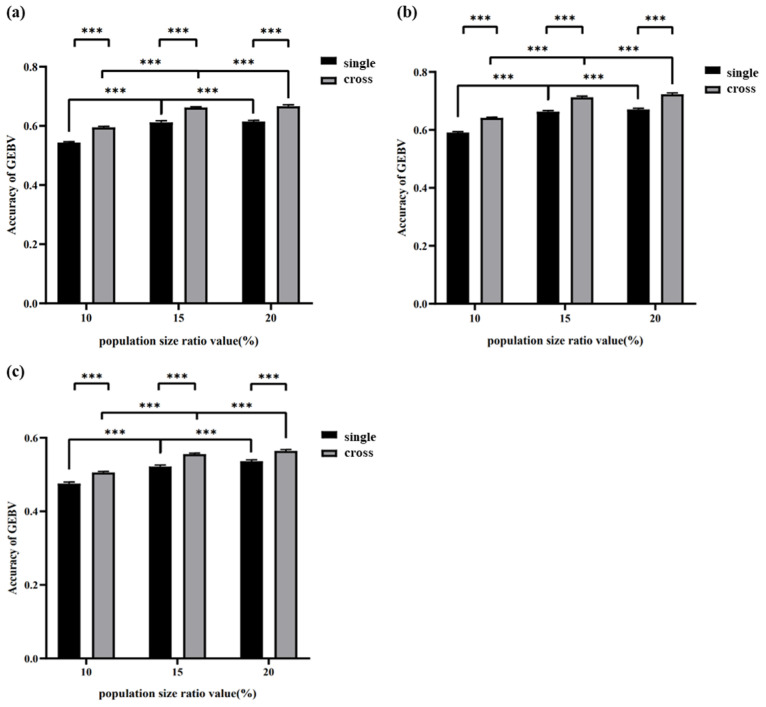
Comparison of GEBV prediction accuracy between the single group (PopB single population) and the cross group (PopA + PopB across populations) under three genomic prediction methods, GBLUP (**a**), ssGBLUP (**b**), and wGBLUP (**c**), when selecting highly genetically similar individuals between PopA and PopB based on Fst. The x-axis shows the percentage of different genetic distance population sizes; the y-axis shows GEBV. *** indicates *p*-value < 0.0001.

This study aims to evaluate the impact of different genetic backgrounds and mixing proportions on the accuracy of GEBV predictions by comparing the accuracy of genomic selection predictions in small populations under different levels of genetic distance between PopA and PopB/C (Figure 4). When analyzing population A combined with different proportions of PopB using three evaluation models, in the subplots of GBLUP (Figure 4a), ssGBLUP (Figure 4b), and wGBLUP (Figure 4c), the GEBV prediction accuracy of the cross group (i.e., the cross-population reference set constructed by mixing PopA and PopB) was significantly higher than that of the signal group (i.e., the single-population evaluation reference set based solely on PopB) at all population size proportion gradients (10%, 15%, 20%). The statistical test results showed that the differences were extremely significant or significant (***, *p* < 0.0001). Meanwhile, as the population size proportion increased from 10% to 20%, the GEBV prediction accuracy of both the signal group and the cross group showed a consistent upward trend. In the GBLUP method (Figure 4a), the accuracy advantage of the cross group over the signal group was sustained. When the population size proportion was 10%, the cross group already showed higher prediction accuracy than the signal group, and as the proportion increased to 15% and 20%, the difference in accuracy between the two groups further widened, with the advantage becoming more pronounced. Under the ssGBLUP method (Figure 4b), the GEBV prediction accuracy of the cross group was also consistently and significantly higher than that of the signal group under all population size proportion conditions. The difference in accuracy between the two groups was clearly evident at the 10% proportion, and as the proportion increased, the accuracy of both groups improved steadily, with the difference remaining statistically significant. For the wGBLUP method (Figure 4c), consistent with the above two methods, the GEBV prediction accuracy of the cross group was superior to that of the signal group at all population size proportions. It is particularly worth noting that when the population size proportion increased from 10% to 20%, the rate of increase in accuracy of the cross group was significantly higher than that of the signal group, resulting in the largest accuracy difference between the two groups at the 20% proportion among all proportion gradients.

**Figure 5:**
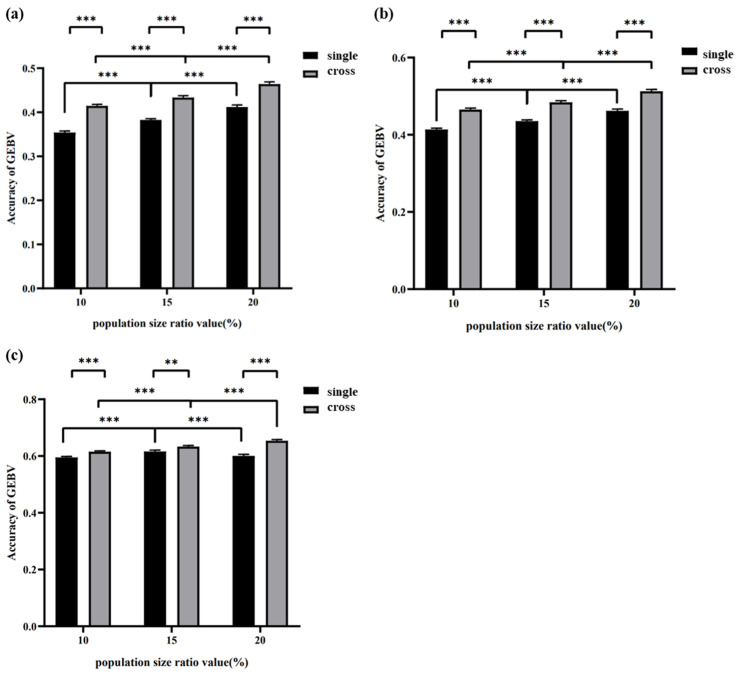
Comparison of GEBV prediction accuracy between the single group (PopC single pop-ulation) and the cross group (PopA + PopC across populations) under three genomic prediction methods, GBLUP (**a**), ssGBLUP (**b**), and wGBLUP (**c**), when selecting highly genetically similar individuals between PopA and PopC based on Fst. The x-axis shows the percentage of different genetic distance population sizes; the y-axis shows GEBV. *** indicates *p*-value < 0.0001, ** indicates *p*-value < 0.001.

When analyzing the cross-composite beef cattle populations formed by supplementing Population A with different proportions of Population C using the three genomic prediction models, the overall trend presented in Figure 5 was analogous to the outcomes obtained from the cross-composite populations of Population A integrated with varying proportions of Population B. Specifically, the GEBV prediction accuracy results obtained using the GBLUP (Figure 5a), ssGBLUP (Figure 5b), and wGBLUP (Figure 5c) methods all showed an upward trend as the population size proportion increased from 10% to 20%. Both the signal group (representing the single-population reference group of PopC) and the cross group (representing the cross-population reference group mixed from PopA and PopC) exhibited increased GEBV accuracy. However, after introducing cross-population individuals with high genetic similarity, the GEBV accuracy of the cross group was significantly higher than that of the signal group at each population size proportion (***, *p* < 0.0001). This indicates that incorporating a corresponding proportion of PopC individuals into the PopA population to form a cross-population reference set can effectively improve the accuracy of GEBV predictions. Moreover, introducing cross-population individuals with high genetic similarity at a proportion of 15–20%, compared to 10%, represents the optimal range for balancing the genetic diversity of the reference set with GEBV prediction accuracy.

Therefore, the results indicate that, compared with constructing single-population reference sets solely based on PopB/C (signal group), introducing corresponding proportions of PopB/C individuals and mixing them with PopA to form cross-population reference sets (cross groups) can effectively enhance the accuracy of GEBV predictions. This enhancement effect is stably present in the three genomic prediction methods—GBLUP, ssGBLUP, and wGBLUP—and shows a further strengthening trend with the increase in population size proportion.

### 3.3. Comparative Analysis of Accuracy Based on GBLUP, ssGBLUP, and wGBLUP Models Under Different Genetic Distance Levels

**Figure 6:**
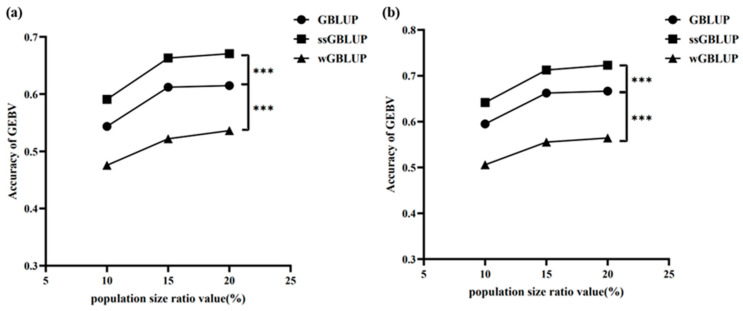
Comparison of Genomic Selection Prediction Accuracy of GBLUP, ssGBLUP and wGBLUP Models for Beef Cattle Populations PopA and PopB under Varying Genetic Distance Gradients (**a**) Accuracy comparison of the single-population reference group (PopB only) across different genetic distance levels between PopA and PopB; (**b**) accuracy comparison of the cross-population reference group (PopA supplemented with different proportions of genetically close individuals from PopB) across different genetic distance levels between PopA and PopB. The x-axis indicates the percentage of individuals representing distinct genetic distance levels, and the y-axis shows the genomic estimated breeding value (GEBV). *** indicates *p*-value < 0.0001.

**Figure 7:**
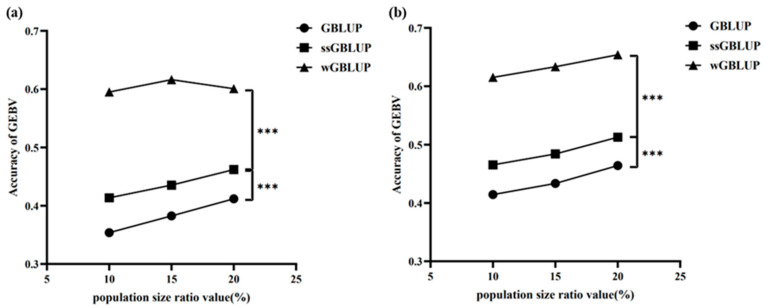
Comparison of the accuracy of three evaluation models, GBLUP, ssGBLUP, and wGBLUP, for PopA and PopC under different levels of genetic distance. (**a**) Comparison of the signal group PopC single-population reference set between PopA and PopC at different levels of genetic distance; (**b**) comparison of the cross group PopA + cross-population reference set with different proportions of highly similar individuals from PopC between PopA and PopC at different levels of genetic distance. The x-axis shows the percentage of different genetic distance population sizes; the y-axis shows GEBV. *** indicates *p*-value < 0.0001.

To evaluate the predictive performance of different genomic prediction methods on GEBV) under single-population (signal) and cross-population (cross) reference set scenarios, we used GBLUP, ssGBLUP, and wGBLUP to assess the accuracy of genomic selection predictions using reference populations constructed by integrating PopA with PopB or PopC at different mixing proportions and genetic distance thresholds. It was observed that the ssGBLUP and wGBLUP methods showed higher accuracy in predicting PopB and PopC, respectively, and there were significant differences compared with the other two methods (Figure 6 and Figure 7). After introducing cross-population individuals with high genetic similarity, the GEBV prediction accuracy was significantly higher than that of the control group using only PopA.

As shown in Figure 6, for the PopB population, whether in the signal group or the cross group, ssGBLUP exhibited the highest GEBV prediction accuracy among the three methods. In the PopB single-population reference set, as the population size proportion increased from 10% to 20%, the accuracy of ssGBLUP rose from approximately 0.60 to around 0.68, showing a clear upward trend. GBLUP also showed an upward trend with the increase in population size proportion, with its accuracy increasing from about 0.55 to 0.62. In contrast, wGBLUP had the lowest accuracy among the three methods, rising from approximately 0.48 to 0.55 as the population size proportion increased. Statistically, there were extremely significant differences in GEBV accuracy between ssGBLUP and GBLUP, and between ssGBLUP and wGBLUP (***, *p* < 0.0001), indicating that ssGBLUP outperformed the other two methods in the signal group (Figure 6a). Moreover, the trend in the cross group was similar to that in the signal group, with ssGBLUP still achieving the highest GEBV prediction accuracy. As the population size proportion increased from 10% to 20%, the accuracy of ssGBLUP rose from about 0.65 to 0.72. The accuracy of GBLUP increased from around 0.60 to 0.68, and that of wGBLUP rose from approximately 0.50 to 0.58. There were also extremely significant differences in GEBV accuracy between ssGBLUP and GBLUP, and between ssGBLUP and wGBLUP (***, *p* < 0.0001), demonstrating that ssGBLUP maintained its advantage in the cross group (Figure 6b). Therefore, under both the signal and cross reference set scenarios, ssGBLUP consistently showed higher GEBV prediction accuracy compared with GBLUP and wGBLUP. Moreover, as the population size proportion increased, the GEBV prediction accuracy of all three methods showed an upward trend, indicating that a larger proportion of individuals in the reference set helps to improve prediction accuracy.

Compared with the PopB population, in the PopC population, whether in the signal group or the cross group, wGBLUP showed the highest GEBV prediction accuracy among the three methods (Figure 7). As the population size proportion increased from 10% to 20%, the accuracy of wGBLUP first rose and then slightly decreased, reaching the highest level of about 0.62 at a population size proportion of 15%. ssGBLUP also showed an upward trend with the increase in population size proportion, with its accuracy rising from about 0.42 to 0.48. In contrast, GBLUP had the lowest accuracy among the three methods, increasing from about 0.36 to 0.42 as the population size proportion increased. Statistically, there were extremely significant differences in GEBV accuracy between wGBLUP and ssGBLUP, and between wGBLUP and GBLUP (***, *p* < 0.0001), indicating that wGBLUP outperformed the other two methods in the signal group (Figure 7a). The trend in the cross group was similar to that in the signal group, with wGBLUP still achieving the highest GEBV prediction accuracy. The accuracy of ssGBLUP increased from about 0.46 to 0.51, and that of GBLUP rose from about 0.41 to 0.47. There were also extremely significant differences in GEBV accuracy between wGBLUP and ssGBLUP, and between wGBLUP and GBLUP (***, *p* < 0.001), demonstrating that wGBLUP maintained its advantage in the cross group (Figure 7b). Therefore, under both the signal and cross reference set scenarios, wGBLUP consistently showed higher GEBV prediction accuracy compared with GBLUP and ssGBLUP. Moreover, as the population size proportion increased, the GEBV prediction accuracy of all three methods showed an upward trend, indicating that a larger proportion of individuals in the reference set helps to improve prediction accuracy.

Furthermore, to further quantify the performance differences among the three methods, Table 2 shows the coefficients (b) and standard errors (SE) of the GBLUP, ssGBLUP, and wGBLUP evaluation models for PopA combined with PopB/C under different levels of genetic distance. The results indicate that wGBLUP has more optimal coefficients and smaller standard errors, demonstrating better adaptability and predictive stability than GBLUP and ssGBLUP in both the PopB and PopC scenarios.

## 4. Discussion

Although across-breed genomic prediction is theoretically capable of improving the accuracy of GEBV (Genomic Estimated Breeding Value) by combining reference populations from different breeds, the accuracy of direct across-breed evaluation is not very high in practical applications [2,27,28,29]. This is mainly due to genetic differences between breeds, inconsistencies in LD (Linkage Disequilibrium) patterns, and differences in allelic substitution effects. The study by Wientjes et al. [8] developed a deterministic formula based on selection index theory, which can accurately estimate the accuracy of across-breed genomic prediction and revealed the impact of the correlation of allelic substitution effects between breeds on prediction accuracy. These findings indicate that in order to improve the accuracy of across-breed prediction, it is necessary to develop new methods to better utilize multi-breed data while overcoming the limitations of existing methods. Therefore, this study innovatively screens for highly differentiated SNPs (single-nucleotide polymorphisms) between populations based on Fst values, calculates Euclidean genetic distances accordingly, and assesses the impact of individuals with different genetic backgrounds at various mixing ratios on the accuracy of GEBV prediction. This approach aims to optimize the construction of across-breed reference sets, thereby improving the accuracy of across-breed genomic prediction. The results show that strategic individual selection based on genetic differentiation patterns can improve the accuracy of across-population genomic prediction.

A key finding of this study is that calculating genetic distances using SNPs screened by high Fst values can effectively capture underlying population structure and highlight genomic regions under selection. By focusing on these highly differentiated sites, we are able to preferentially select individuals from PopB and PopC that have the highest genetic affinity with PopA. This method provides a principled way to minimize the negative impact of population structure on genomic prediction—which usually stems from differences in allele frequencies and linkage disequilibrium (LD) patterns between populations. Studies have shown that there are significant differences in the LD patterns of SNPs and QTLs between different breeds, and simply combining reference populations does not effectively improve prediction accuracy. However, by selecting SNPs with high LD phase consistency, prediction results can be significantly improved, indicating that LD phase differences are a key factor affecting the accuracy of multi-breed genomic prediction [30]. In addition, differences in QTL allele frequencies between breeds can lead to different QTL substitution effects. Even if the QTL effects are the same across breeds, differences in allele frequencies will result in different substitution effects. These differences not only affect prediction accuracy but also indicate that in multi-breed genomic prediction, it is necessary to take into account the differences in allele frequencies and LD patterns to optimize the prediction model [31,32]. Therefore, the use of Euclidean distance as a measure of genetic similarity in this study becomes a robust criterion for individual selection, thereby facilitating the more coordinated integration of exotic germplasm into the reference population.

When the reference population included the 10–20% most similar individuals from PopB or PopC, the prediction accuracy of GEBVs (Genomic Estimated Breeding Values) was significantly improved compared with our previous study results based on multidimensional scaling analysis (MDS) to calculate the genetic distance between PopA and PopB/C separately [33]. This result highlights the key role of genetic proximity between the training population and the prediction population in genomic prediction. Even if there is a large genetic distance between the original populations, constructing a reference population by screening individuals with high genetic similarity can still effectively improve prediction accuracy. In addition, the results of introducing PopB/C into PopA at different proportions and constructing mixed reference populations (with the highest mixing proportion up to 20%) show that the proportion and genetic composition of individuals in the reference population jointly affect prediction accuracy. Specifically, as the proportion of PopB/C individuals in the mixed reference population increases, the prediction accuracy of GEBVs shows an upward trend. This indicates that when constructing a reference population, appropriately increasing the proportion of individuals from PopB/C can provide richer genetic information for the prediction model, thereby helping to improve prediction accuracy.

In addition, this study also found that the optimal mixing proportion varies depending on the source population. For PopB, ssGBLUP (single-step Genomic Best Linear Unbiased Prediction) always showed higher prediction accuracy of GEBVs (Genomic Estimated Breeding Values), and as its proportion in the reference population increased, the prediction accuracy of all three methods showed an upward trend. This indicates that increasing the proportion of PopB individuals helps to improve prediction performance. Conversely, for PopC, wGBLUP (weighted Genomic Best Linear Unbiased Prediction) always had higher prediction accuracy, and as the proportion of PopC increased, the accuracy of all methods also showed an upward trend. It should be noted that although increasing the mixing proportion of a certain population generally helps to improve accuracy, introducing too many distantly related individuals beyond a certain threshold may introduce noise and reduce prediction performance. This was clearly verified in the mixed scenarios designed in this study. The above results are consistent with previous studies, that is, in multi-population or across-breed genetic evaluation, the shared genomic fragments retained due to common ancestry or convergent selection have important information value for across-population breeding value prediction. These fragments usually reflect the genetic differentiation and adaptive characteristics between populations, and therefore show strong prediction ability in GS (Genomic Selection) [20,34,35]. Secondly, this study further emphasizes that when constructing a mixed reference population, it is necessary to seek a fine balance between expanding genetic diversity and maintaining the compatibility of LD (Linkage Disequilibrium) structure and allele frequencies between the reference population and the target population. The study by Zhang et al. [36] also shows that the unique genetic background and historical differentiation process of each population play key roles in determining the composition of the ideal reference population. The genetic characteristics and differentiation history of different populations lead to significant differences in their contributions to the prediction model. Therefore, when constructing a mixed reference population, one should not simply pursue the increase in the number of populations, but should systematically evaluate the genetic characteristics of each population to determine the optimal mixing proportion, thereby improving the accuracy and robustness of genomic prediction.

However, despite the fact that the method of screening highly differentiated SNPs based on Fst and calculating Euclidean genetic distance showed higher prediction accuracy in this study, as Chang et al. [15] pointed out, the Fst method, while having the advantage of identifying population differentiation characteristics, also has certain limitations. For example, the method mainly relies on population structure information and does not fully consider the redundancy between SNPs, which may lead to overlapping information sites in the selected characteristic SNP set, thereby reducing the overall efficiency of the screening. In addition, Fst screening tends to retain highly differentiated sites and may miss some SNPs with lower differentiation between populations but with important contributions to the target trait, which to some extent affects the completeness of the captured genetic information. On the other hand, the calculation process of Fst is relatively complex, requiring accurate estimation of the genetic structure of populations and the distribution of allele frequencies. Therefore, in practical applications, it is easily restricted by factors such as sample size and data quality.

## 5. Conclusions

This study systematically evaluated the efficacy of Fst-mediated highly differentiated SNP screening in cross-population genomic prediction and analyzed how genetic background and mixing ratio affect prediction accuracy via a multi-level mixing design. The results demonstrated that reference sets constructed using genetic differentiation information significantly enhance the accuracy and robustness of cross-population genomic selection (GS). It was confirmed that rational mixing ratio regulation and appropriate statistical model matching are key to optimizing prediction performance, which requires concurrent consideration of population origin and genetic similarity. The study identified three core optimization elements for cross-breed genomic prediction: population genetic distance, reference set mixing ratio, and prediction model selection. This strategy provides theoretical support for the efficient utilization of cross-population genetic information in resource-limited local breeds and lays a foundation for developing adaptable and scalable genomic selection schemes. Despite the universal implications of the conclusions, several issues warrant further investigation, including the impact of genotype-by-environment interaction (G × E) on allele effect stability and the applicability of Fst-based screening for low-heritability or strongly selected traits. Future research could integrate multi-omics data (e.g., transcriptomics, epigenomics) and adopt advanced modeling approaches (e.g., Bayesian hierarchical models, machine learning algorithms) to optimize cross-population prediction models across broader genetic backgrounds and environmental scenarios.

## Figures and Tables

**Table 1:** Summary of population structure and simulation parameters.

Parameter	PopA ^1^	PopB ^2^	PopC ^3^
Step 1: Historical Generations (HG)			
Phase 1 generation count (population size)	0 (200)	0 (500)	0 (1000)
Phase 2 generation count (population size)	1000 (200)	1000 (200)	1000 (1000)
Phase 3 generation count (population size)	1095 (1000)	1095 (1000)	1095 (200)
Step 2: Recent Generations (RG)			
Number of male founders from HG	200	220	200
Number of female founders from HG	2800	2335	2800
Selection and mating	ebv/h		
Sire/Dam replacement	0.6/0.3	0.5/0.3	0.5/0.2
Mating strategy	Random		
Culling scheme	ebv/L		
Genome			
Chromosome count	29 (no X Chr)		
Genome length	2715.85 cM		
Number of markers	100 k		
Marker/QTL genomic locations	Random		
Marker/QTL allele count	2/2 3 4		
Marker allele frequency distribution	Equal		
QTL allelic effect sizes	Equal		
Additive allelic effects of QTL	Gamma distribution (shape = 0.4)		
Marker genotype missingness proportion	0.01		
Marker genotyping error rate	0.005		
Recurrent mutation frequency	0.0001		

**^1^** Population A; **^2^** Population B; **^3^** Population C.

**Table 2:** Comparison of coefficients (b) and standard errors (SE) of the GBLUP, ssGBLUP, and wGBLUP evaluation models for PopA and PopB/C under different levels of genetic distance.

Pop	Percentage	GBLUP		ssGBLUP		wGBLUP	
		b ^1^	SE ^2^	b	SE	b	SE
PopB	10%	1.12	0.022	0.96	0.026	1.18	0.06
	15%	0.93	0.042	1.03	0.003	0.85	0.02
	20%	1.23	0.021	1.23	0.064	1.39	0.017
PopC	10%	1.22	0.021	1.19	0.002	0.86	0.016
	15%	1.43	0.019	0.96	0.057	1.29	0.015
	20%	1.34	0.017	1.24	0.054	1.15	0.014

^1^ Unbiasedness; ^2^ standard error of the estimate.

## Data Availability

The data analyzed in this study were generated through simulation and are not deposited in a public repository. Detailed descriptions of the simulation parameters and procedures are provided in Section 2 of this manuscript. The simulation was conducted using publicly accessible software, which can be found at https://animalbiosciences.uoguelph.ca/~msargol/qmsim/ (accessed on 11 June 2019). Upon reasonable request, the corresponding author will provide any additional data necessary for the replication of the study’s findings.

## Abbreviations

The following abbreviations are used in this manuscript:

GS	Genomic selection
TBV	True breeding value
EBV	Estimation of breeding value
BLUP	Best linear unbiased prediction
GEBV	Genomic estimated breeding value
SNP	Single-nucleotide polymorphism
MAF	Minor allele frequency
LD	Linkage disequilibrium
QTL	Quantitative trait loci
GBLUP	Genomic best linear unbiased prediction
ssGBLUP	Single-step genomic best linear unbiased prediction
wGBLUP	Weighted best linear unbiased prediction
GRM	Genomic relationship matrix
Fst	Fixation index
