# Correction: Zhou et al. Leveraging Fst and Genetic Distance to Optimize Reference Sets for Enhanced Cross-Population Genomic Prediction. *Animals* 2026, *16*, 359

**DOI:** 10.3390/ani16111741

**Published:** 2026-06-05

**Authors:** Le Zhou, Lin Zhu, Fengying Ma, Mingjuan Gu, Risu Na, Wenguang Zhang

**Affiliations:** 1 College of Animal Science, Inner Mongolia Agricultural University, Hohhot 010010, China; zxcvbnm8880314@163.com (L.Z.); zhulinynacxhs@163.com (L.Z.); fengyingma1997@163.com (F.M.); gmj0119@yeah.net (M.G.); narsanjargal@imau.edu.cn (R.N.); 2 Inner Mongolia Engineering Research Center of Genomic Big Data for Agriculture, Hohhot 010010, China; 3 College of Life Science, Inner Mongolia Agricultural University, Hohhot 010010, China

## Error in Figure and Legend

In the original publication [1], there was a mistake in the legend for Figures 4 and 5. Spelling errors were present in the words of the legends due to our oversight during manuscript preparation. The corrected legend appears below. 

In the original publication, there was a mistake in Figures 4 and 5 as published. Spelling errors were present in the annotations/labels within the figures due to our oversight during manuscript preparation. The corrected Figure 4 and Figure 5 appear below. 

## Additional Affiliation(s)

In the published publication, there was an error regarding the affiliation(s) for all authors. In addition to correcting Affiliations 1 and 2, the Affiliation 3 for author Wenguang Zhang has been added. The updated affiliation(s) should include: ^1^ [College of Animal Science, Inner Mongolia Agricultural University, Hohhot 010010, China; zxcvbnm8880314@163.com (L.Z.); zhulinynacxhs@163.com (L.Z.); fengyingma1997@163.com (F.M.); gmj0119@yeah.net (M.G.); narsanjargal@imau.edu.cn (R.N.)], ^2^ [Inner Mongolia Engineering Research Center of Genomic Big Data for Agriculture, Hohhot 010010, China], ^3^ [College of Life Science, Inner Mongolia Agricultural University, Hohhot 010010, China]. 

The authors state that the scientific conclusions are unaffected. This correction was approved by the Academic Editor. The original publication has also been updated.

## Figures and Tables

**Figure 4:**
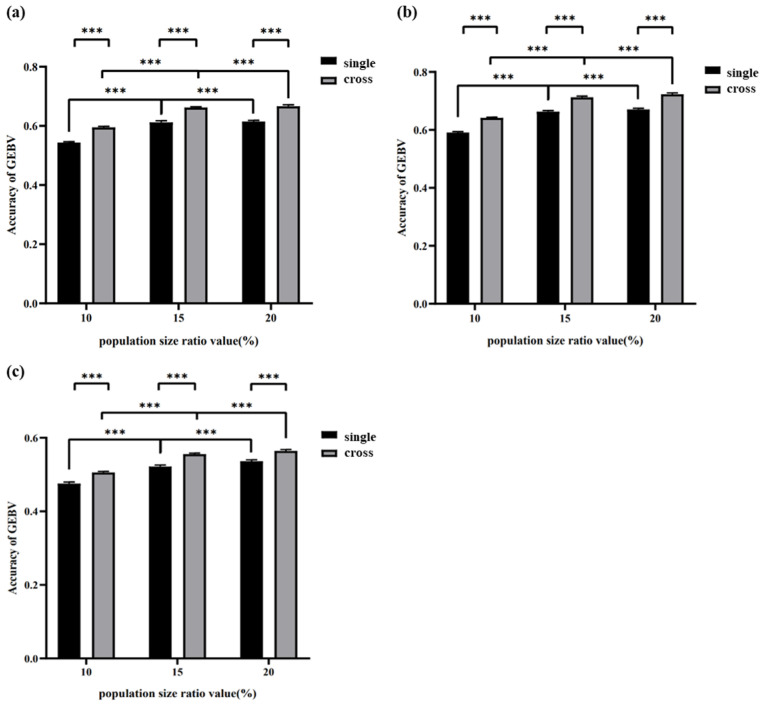
Comparison of GEBV prediction accuracy between the single group (PopB single population) and the cross group (PopA + PopB across populations) under three genomic prediction methods, GBLUP (**a**), ssGBLUP (**b**), and wGBLUP (**c**), when selecting highly genetically similar individuals between PopA and PopB based on Fst. The x-axis shows the percentage of different genetic distance population sizes; the y-axis shows GEBV. *** indicates *p*-value < 0.0001.

**Figure 5:**
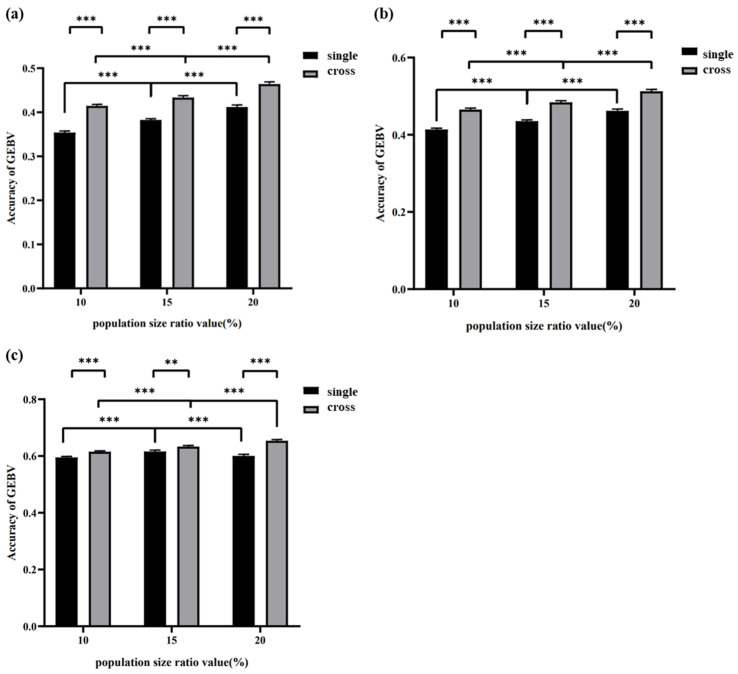
Comparison of GEBV prediction accuracy between the single group (PopC single pop-ulation) and the cross group (PopA + PopC across populations) under three genomic prediction methods, GBLUP (**a**), ssGBLUP (**b**), and wGBLUP (**c**), when selecting highly genetically similar individuals between PopA and PopC based on Fst. The x-axis shows the percentage of different genetic distance population sizes; the y-axis shows GEBV. *** indicates *p*-value < 0.0001, ** indicates *p*-value < 0.001.
